# Treatment of Blenorrhœa and Kindred Affections

**Published:** 1861-03

**Authors:** 


CHICAGO

### MEDICAL JOURNAL.

VOL. IV.] MARCH, 1861. [NO. 3.

### SELECTED.

### TREATMENT OF BLENORRHŒA AND KINDRED AFFECTIONS.

Dr. Morey employed in four cases of gonorrhœa: twenty grains of Herrings & Co.'s extract of India hemp, mixed with a half-a-drachm of sugar of milk, and divided into sixty powders, one of which is to be taken every three or four hours. The success was complete in every case.—*Ogleth. Med. and Surg. Journ.*

The internal use of hemlock extract (*conium maculatum*), as recommended by Dr. Staats, of Albany, has been found of good effect in several cases of acute gonorrhœa, by Dr. A. H. Stephens, of Camden, Ohio. In two cases the lunar caustic, zinc, copaiba and cubebs had failed to suppress the discharge, while the extract of hemlock, given in the form of pills, twelve grains every two hours, proved sufficient for a complete cure in a few days. Where the inflammatory symptoms are very prominent, the treatment is commenced with calomel and compound powder of jalap.—*St. Joseph Jour. of Med. and Surg.*

A combination of vinum colchici and tincture of opium, internally exhibited for a rheumatic conjunctivitis, by Dr. Eisenmann, of Werzburg, turned out accidentally as a cure for a coexisting blenorrhagia. Further experiments by Dr. Eisenmann himself, and Dr. Collin, of Dresden, have since established the value of that combination against all blenorrhagic discharges. The mixture employed consists of twelve grammes of vinum colchici and two grammes of laudanum. The dose is eighteen or twenty drops, three times a day. Milk was ordered as the principal article of food, and absolute rest was enjoyed. Externally, only frequent applications of tepid water. All cases thus treated were cured within a week, and most of them in a few days.—*Bull. Gen. de Therap.*; *Brit. and Foreign Med.-Chir. Rev.*

Dr. Diday employs (*Annuaire de la Syph*, etc.,) in chronic blenorrhœas injections of nitrate of silver into the prostatic glands of the urethra. To effect this, he first measures the urethra by introducing an elastic catheter and gently drawing it back during the flow of the urine. As soon as this ceases, the end of the catheter is in the prostatic portion; at that moment, then, the instrument is marked at the point in contact with the external orifice, and about an hour afterwards the injection is made. For this purpose, the patient stands before the surgeon, holding the penis in a horizontal direction; the catheter being introduced, a solution of about five or ten grains of nitrate of silver in about an ounce of water is injected with a glass syringe, at first one-fourth of this quantity being used. In intervals of half-minutes the catheter is drawn back about two lines and another portion of the solution injected. This may be repeated in thirty-six or forty-eight hours; the abnormal secretion usually ceases in half that time. —*Vierteljahrschr. f. Prakt. Heilk*, Bd. 66.

In acute cases, where only the anterior portion of the urethra is affected, the same author found (*Gaz. Med.*, 1859, 26) one injection sufficient, provided the urethra be previously washed out with water. Three parts of the nitrate are required to one hundred and eighty parts of distilled water, of which solution two drachms should be injected at a time, but not beyond a distance of two inches. The fluid is moved up and down in the urethra by pressure with the fingers for about three minutes, and then allowed to escape. That constitutes the whole treatment.—*Ibid*, Bd. 65.

The alcoholic tincture of aloes (about five drachms diluted with five ounces of water) has been employed successfully against blenorrhœa by S. Gamberini, of Boulogne, and others. Three injections are made every day. The discharge disappears in less than a fortnight.—*Revue de Therap; Jour. of Mat. Med.*

Repeated trials have convinced Dr. Gaby, of Paris, that injections of oxyde of bismuth constitute the very best treatment of gleety discharges. Thirty parts are suspended in two hundred of rose water, and so injected as to leave as large a deposit of the salt as possible in the canal. Three injections per day should be employed at first, and then fewer. Urethral discharges unconnected with gonorrhœa, as observed in certain diatheses, in masturbation, venereal excesses, etc., have been successfully treated in this manner. Balanitis, balano-posthitis and herpes præputialis yield rapidly to bismuth applied in powder, after cleansing the part and then covering with cotton. The various forms of vulvar leucorrhœa may be treated with the same agent; after removing the complications, the bismuth acts upon the discharge like a specific. But it is to be remembered that this remedy is only adapted to chronic cases; pain and other signs of acute inflammation contraindicate its employment.—*North Amer. Med. Reporter.*

Dr. Weeden Cook endeavors to affirm that copaiba in the treatment of gonorrhœa is not only unnecessary, but in a great many instances injurious. In six thousand cases that have come under the author's care at the Royal Free Hospital, he took advantage of the large opportunity thus offered, to test the different methods of treatment which had been suggested, and now makes the following statements in regard to them. The abortive treatment by strong injections of nitrate of silver has sometimes resulted in inflammation of the bladder, and has proved a failure. Dilutants are slow in their action and not readily employed by persons in active business. Diuretics are scarcely more successful, and the administration of saline aperients is generally attended with an aggravation of the ardor urinæ as well as chordee. The most successful treatment is that by the alkaline carbonates, given with a view of neutralizing the acid in the urine, and thus removing one great source of irritation from the inflammed urethra, whereupon the subsidence of the inflammation is effected by nature. As auxiliaries, especially where there is œdema of the prepuce, lead lotions and elevation of the penis against the abdomen, are recommended. When the inflammation has subsided, and a muco-purulent discharge is left, the speediest cure is effected with injections of chloride of zinc, one, or better two, grains to one ounce of water. In the strumous, the dyspeptic, those of dissipated habits, and old-offenders, the alkaline carbonates are not called for, because either the urine is not acid, or the inflammation does not run high. In such cases, the tincture of iron or sulpheric acid and bark, or gentian, or calumba, may be advantageously employed from the commencement. The chloride of zinc is in such cases of the utmost value. Respecting diet, scarcely any restrictions need be enforced after the subsidence of the inflammatory symptoms; beer or wine in moderate doses may be advantageously used by those accustomed to these beverages.—*London Lancet.*

A very specified treatment of gonorrhœa and gleet has been given in the lectures on venereal diseases, by Dr. F. J. Bumstead, published in the late *New York Journal of Medicine.* For the abortive treatment of the disease in the male adult, he prefers the weak to the strong solution of nitrate of silver, say one or one and a half grains in six ounces of water. This should be injected at short intervals, until the discharges become thin and watery, and slightly tinged with blood. But this treatment is only adapted to the first stage of the disease. When acute inflammatory symptoms have already set in, or the patient suffers from scalding in passing water, a brisk cathartic is the first thing to be given. If the penis is much swollen, a mixture is advised of bicarbonate of potassa, two drachms; tincture of hyosciamus, one ounce; mucilage of gum Arabic, five ounces; a tablespoonful every three hours. As soon as a syringe can be inserted without much pain to patient, an injection is made after every passage of urine with a solution of one scruple of extract of opium in one ounce of glycerine and three ounces of water. In subacute cases, from half to one grain of sulphate of zinc to the ounce of water may be added. As a local means for the relief of uneasiness, local pain, scalding in micturition, Dr. Bumstead fully endorses Dr. Milton's statements in regard to hot water, as hot as it can be borne. For the third stage of gonorrhœa, injections are pronounced a weapon indispensable to the surgeon. Sulphate of zinc (twelve or more grains in four ounces of water) and nitrate of silver constitute the proper ingredients for these injections. In connection with them, copaiba or cubebs may be administered; the last named either alone or with the former, or, if the case demands a tonic, with iron or quinine. For the relief of the chordee, either lupulin will serve in fifteen grain doses, or full doses of camphor tincture, or lactucarium and camphor, one grain of each, made into a pill.

In the treatment of gleet, the bowels should be kept open daily with pills composed of half a grain of strychnine and half a drachm of the compound colocynth pill; this to be made into thirty pills, one of which is to be taken at bedtime. As a tonic and astringent, the muriated tincture of iron is usually indicated, and best exhibited in doses of from five to twenty drops three times a day. The tincture of cantharides may, where it is indicated, be combined with the iron tincture, in the proportion of one to three, and ten drops of such combination will form a dose. Where the constitutional impairment is considerable, quinine may be added. In regard to local treatment, Dr. Bumstead speaks highly of bougies. Except when aggravating the symptoms, they may be passed every second or third day at first, and afterwards every day, or even twice a day. They may also be medicated with gray ointment and extract of belladonna. For injections, sulphate of zinc is preferable, two or three grains to the ounce of water forming the standard of medium strength. This solution should be employed as frequently as the patient is able to pass his water, or every two or three hours. In obstinate cases blisters may be tried.—*Lancet & Observer.*

